# The Importance of “Easy Japanese”: Communicating Health Information to Foreigners in Japan

**DOI:** 10.7759/cureus.27036

**Published:** 2022-07-19

**Authors:** Remi Oi, Ryuichi Ohta, Yukiko Shiba, Chiaki Sano

**Affiliations:** 1 Family Medicine, Shimane University Faculty of Medicine, Izumo, JPN; 2 Communiy Care, Unnan City Hospital, Unnan, JPN; 3 Family Medicine, Unnan Toiro, Unnan, JPN; 4 Community Medicine Management, Shimane University Faculty of Medicine, Izumo, JPN

**Keywords:** medical school students, foreign residents in japan, health communication, healthcare access, japan, easy japanese

## Abstract

The number of foreign workers in Japan has been increasing in recent years. In Shimane Prefecture, people from non-English speaking countries account for most of the foreign resident population. Language barriers pose numerous challenges for this population. Their problems communicating in the medical context, in particular, contribute to their avoidance of hospitals. In addition to translation machines and English, "Easy Japanese" has been found to help Japanese healthcare workers communicate with foreign patients. “Easy Japanese" refers to easy-to-understand Japanese that involves rephrasing words and sentences. The use of Easy Japanese should be promoted among medical professionals in Japan as it is considered a communication skill that can be improved through practice. A voluntary study group was formed among medical students. During the first session, students were presented with background information, explaining why the need for Easy Japanese is increasing. In the second session, they practiced paraphrasing words. Finally, in the third session, they conducted simulated medical communication and practiced Easy Japanese with foreign residents to determine whether they were able to convey their intentions. Participants were recruited via social networking service, with five participants in the first session, five in the second, and eight in the third. Through this project, it became clear that for participants, the usual way of speaking Japanese came first in practice and that it was difficult for them to produce easy-to-understand phrases at the spur of the moment without practice. Additionally, medical students reported that the expressions they acquired through several practice sessions were helpful when talking with international students on campus. The final session involved a student-led Easy Japanese study group. Based on the students’ comments, we found that this study group was useful for them. Accordingly, Easy Japanese education should be continued and expanded to more students in the medical field and to the local community, including foreign residents, to measure its effectiveness.

## Introduction

Communication challenges in obtaining healthcare, where language barriers are the primary cause, are found worldwide [1,2]. One aspect of that language barrier, and one of the factors that can limit foreign residents' access to healthcare, is created by the documentation and registration systems within healthcare systems. In Europe, barriers to patient safety have been found in areas such as nursing care, medication, diagnosis, and communication between doctors and patients in acute situations [1]. The need to remove language barriers by providing appropriate communication bridges in situations where interpreters are not available is also discussed in the literature [1]. Other cultural factors can present additional barriers. For example, Somali women in Minnesota were less likely to seek cancer screenings, and culturally appropriate interventions needed to be implemented through proper communication to improve this issue [3]. Again, bridging the language barrier is critical.

According to a Japanese Ministry of Health, Labor, and Welfare survey, the average number of foreign patients per hospital has increased in the last three years [4]. In Japan, as in other parts of the world, there is a general problem involving the medical community's lack of contact with residents due to such residents' difficulties in accessing the information on cultural exchange events, their inability to carry out administrative procedures on their own and challenges in job hunting. Moreover, at medical institutions and health centers, communication problems are the most frequently cited problem by foreign patients. Many have varying degrees of Japanese language ability. This is where most healthcare disparities among foreigners stem from; such language barriers are considered the root of the health disparities among foreign residents in Japan [5]. Owing to the diversity of language among foreigners and the lack of an established system, the medical interpreting services being provided are inadequate. Problems arise when medical professionals search for the most appropriate hospital given a patient's condition; when patients attempt to explain their medical conditions using questionnaires, including those with open-ended questions; and when doctors do not provide sufficient explanations about illnesses and treatments [5]. Although the health care access problems foreigners faced differ according to their Japanese language proficiency levels, healthcare workers' use of "Easy Japanese" and expressions familiar to both foreigners and Japanese people when treating foreign patients have led to such patients' improved health literacy [5,6].

In a Japanese national survey, those experiencing communication difficulties were 13.1% among foreign residents in Japan [7]. These were part of the Technical Internal Trainee Program - a human resources program aimed at candidates in developing countries to acquire skills that are unavailable or difficult to acquire in their home countries [7,8]. For example, in Shimane Prefecture, where the study was conducted, 3,435 of the 8,917 foreign residents are from Brazil, and more than 86% are from non-English speaking countries [7]. Problems faced by foreign residents include language barriers that contribute to their avoidance of hospitals, treatment delays, inability to understand the results of medical examinations, and failure to take prescribed medication correctly, which can impact the effectiveness of treatment and lead to accidents. For example, according to a Brazilian resident of Izumo City, Shimane Prefecture, the location of the university campus, many Brazilians living in Japan do not speak Japanese. Therefore, the financial burden of hiring a Japanese interpreter is significant, as they can earn around 200,000-300,000 yen a month for 14 hours of work a day, four days a week.

Easy Japanese is a communication skill that can be used to communicate with foreigners in Japanese [9]. This is a type of communication method proposed by Kazuyuki Sato in the wake of the Great Kanto Earthquake to convey disaster information "quickly," "accurately," and "concisely" to foreign victims [10]. Today, Easy Japanese is being adopted in tourism, the press, and official texts. It is a simple, easy-to-understand way of speaking Japanese that involves simplifying expressions. It is a method of rephrasing technical terms, honorifics, and idiomatic phrases to explain the pain or condition. Key points include keeping the ends of sentences brief, focusing on the main point, and not using onomatopoeia. Medical professionals can use Easy Japanese to facilitate communication with foreign patients and help build their trust [6].

To promote the use of Easy Japanese in the future, it is necessary to establish educational venues and methods on how to use it. In particular, there is an urgent need for education on Easy Japanese in Japanese medical settings, where foreigners face difficulties [11]. There are learning environments in Japan regarding Easy Japanese. In the learning environment, students can understand that the number of foreign residents in Japan is increasing. Students can get interested in the Easy Japanese communication method through learning. In learning Easy Japanese, they can learn how valuable the skill is by directly listening to practitioners engaged in multicultural activities. Additionally, activists can support the daily lives of foreigners by accompanying them when they go to the city hall or hospitals. They can then plan and conduct study sessions to convey this knowledge to as many other medical students as possible [11]. In this paper, we describe the concrete ways of this educational intervention of Easy Japanese in rural contexts and the learning contents of students in the intervention.

## Technical report

All meetings called for one facilitator, who is engaged in multicultural activities in Unnan City, next to Izumo City. Specifically, she supports the daily lives of foreign residents by accompanying them when they go to the city hall or hospitals. Each session lasted about two hours, including Q&A. The book "Easy Japanese for Medical Professionals" was used as teaching material for the study sessions. The study session was announced via social networking services (SNS) to students and faculty members of Shimane University School of Medicine, and participants were invited (Table 1).

**Table 1:** The characteristics of the cases SNS: social networking services

Case	Content	Notification method	Format	Participants
Case 1	Background on the growing need for Easy Japanese; what is Easy Japanese?	SNS	online	Five 2nd to 5th-year medical students (two males, three females), Shimane University School of Medicine, and one instructor
Case 2	Hands-on practice; paraphrasing words	SNS	face to face	Five 2nd to 4th-year medical students (three males, two females) at Shimane University School of Medicine, one professor, and one instructor
Case 3	Hands-on practice; paraphrasing sentences	SNS	face to face	Six 1st to 5th-year medical school students (three males and three females), Shimane University School of Medicine, two foreign residents (one male and one female), and one instructor

Case 1

In the first session, the lecturer, who is engaged in multicultural activities, talked about the current increase in foreign residents and what she experiences when interacting with them. Through the talk, the five participants were persuaded that healthcare workers’ use of Easy Japanese during health communications can be beneficial to foreigners and all other groups of residents.

After the study session, we asked for feedback in Japanese. One participant stated, “When you go to a hospital, you surely have some kind of physical or mental problem, and that alone is stressful, but on top of that, I think it’s challenging to encounter a language barrier during medical treatment. My feeling is that this gentle form of the Japanese language can break down this language barrier little by little and [Easy Japanese] is an essential and crucial skill that all medical professionals should acquire.”

Similarly, another participant stated, “I realized that this applies outside the medical field. It is the way we deal with people that makes them feel that they can talk with or to this other person. So I think the concept of Easy Japanese is essential in all situations, not just language barriers.”

Participants learned that in addition to gestures, smartphones, and translators, staff should use Easy Japanese when conversing with patients in the field to elicit and convey accurate information that would lead to the appropriate diagnosis.

Case 2

In the second session, the six participants, including one professor and two new participants, exchanged ideas on how to paraphrase everyday words such as "drinking" and "living alone" and medical jargon such as "prescription" and "poultice". Paraphrases for "drinking" included “drinking alcoholic beverages", and "living alone" included "living alone in one's own home" or "living by oneself". Some participants suggested that "prescription" could be expressed as "paper" depending on the context and that "poultice" would be easier to convey by showing the patient a picture of the actual product or using a translator. Participants felt that they learned a lot from the session.

After the study session, we asked for feedback in Japanese. One participant stated, "In addition to learning the philosophy and tips from the Easy Japanese language class, listening to other students’ various ideas and opinions gave me the opportunity to think about what Easy Japanese is and what is important in my way of communicating with others. I realize that it is a useful skill to have when dealing with patients, whether they are foreigners or Japanese.”

Another participant stated, “It was through the Easy Japanese class that I became aware for the first time of some of the difficulties involved in my native language, Japanese, and I thought carefully about how to change the words that I usually use into easy expressions that anyone can understand. This led to the discovery that certain words can also be taken as complex. I am delighted to have learned about the Japanese language through the Easy Japanese class.”

The participants felt that continuous practice would be necessary to acquire communication skills in Easy Japanese and that they could use these skills during health communications with patients of all ages, including the foreigners who they are expected to be involved with in practicing community medicine.

Case 3

In the third session, two foreigners working as foreign language teaching assistants at Japanese elementary and junior high schools in the same town were invited to participate and play the role of patients. The two foreigners were introduced by the lecturers invited to the first lecture. With four first-time participants and the same instructor we called the first time, the participants reviewed the main points of Easy Japanese. Then, they practiced communicating with the teaching assistants, simulating events that might occur during a medical examination, such as explaining a disease or prescribing medicine. The eight participants were divided into four two-member teams, and each team collaborated to paraphrase the sentences on the agenda. Then, each team presented the Easy Japanese expressions they created to the two foreign teaching assistants simultaneously. The medical students asked them if they could understand and also asked them to explain what they were expected to do, i.e., the simulated treatment they were to follow, after the communication to see if their expressions were understood. If they were not, the medical students patiently rephrased their ideas on the spot or used simple English or illustrations.

After the study session, we asked for feedback in Japanese. One participant stated, “Many foreigners living in Japan can speak Japanese well enough for daily life. I used to think that English or their native language would be their only primary means of communication, but now I realize that Easy Japanese can be a more excellent skill than we imagined.”

Another participant, who has an American relative, stated, “Although I used to speak Japanese only to my father, who clearly understands what level of Japanese he understands and what level he doesn't understand, and it was good, I realized that I have to explore each person's language status and adapt to each individual. I used to try to force myself to speak English when I met a foreigner for the first time, but I realized that my poor English could be misinterpreted, which I felt could be a problem when providing medical care and could even hinder it.”

The teaching assistant from the Philippines who participated in the practical training as a patient stated, “It was an opportunity for me to confirm how to use my limited Japanese. I also felt less resistance toward the hospital through my interactions with the medical students.”

The participating medical students learned that to provide safer and more secure medical care, they need to be flexible and decide whether to use Easy Japanese, standard Japanese, or another language. They should do this depending on the language preferences and Japanese ability of their conversation partner while exploring their situation through dialogue.

## Discussion

These study sessions involved Easy Japanese methods for medical students. Through all three sessions, three lessons were learned. First, medical professionals need to acquire a wide range of communication skills. Second, the Easy Japanese method is one of the new communication skills being used in the field of medicine. Third, specific communication skills with foreigners should be kept in mind when using Easy Japanese in actual medical practice.

Now and in the future, medical professionals need communication skills such as Easy Japanese to provide seamless medical care in multicultural and multilingual societies. The 2016 Report of the Survey of Foreign Residents in Japan reported that more than 70% of foreign residents could converse to some extent in Japanese [12]. However, while Easy Japanese is becoming more widely used in the government and media from the perspective of disasters and tourism, it is reportedly rarely used in medical institutions because of the lack of knowledge among health care professionals [6]. The lecture by the volunteer involved in multicultural activities in Session 1 helped participants deepen their understanding of the lack of widespread use of Easy Japanese in medical institutions despite the increasing number of foreign patients who speak the language.

Medical professionals must understand the principles of Easy Japanese and use them appropriately to facilitate their proper use in practice. The principles of Easy Japanese include: (1) focusing on information of high importance, (2) avoiding ambiguous expressions, (3) rephrasing complex vocabulary, (4) using easy words, and (5) simplifying sentence structure [9]. For example, rephrasing "drinking" to "drinking alcoholic beverages" or "living alone" to "living alone in one's own home" or "living by oneself" in study groups fall under the third principle. Besides, some participants stated that "prescription" can be expressed as "paper" at medical institutions' convenience. However, this expression could be misunderstood by foreigners because the expression is too simple. To mitigate a potential misunderstanding, medical professionals should use simple and concrete phrases with their actual names like “this paper, a prescription.”

To facilitate communication with foreign patients in Japan, medical professionals must consider the peculiarities of the language. The use of honorifics and onomatopoeia to describe pain in Japanese have been pointed out as sources of difficulty when attempting to use Easy Japanese in medical institutions [6]. Regarding the former, through the workshop, we observed that Japanese speakers use idiomatic expressions in daily conversation. To be attentive to patients, they tend to use polite but unkind phrases that are difficult to understand. Regarding the latter, participants learned that the use of numbers can be effective in discussing levels of pain without onomatopoeia. Through the dialogue, the participating medical students realized that they needed to deepen their understanding of cultural differences across the language chain, such as how honorifics are used and the presence or absence of onomatopoeia.

Foreign residents’ participation in the workshop was expected to promote mutual understanding and to provide a place where the foreign participants, whose interactions were expected to contribute to the medical students’ learning, could experience "symbiosis” with medical professionals [6]. The impressions reported by the foreign residents who participated in the workshop revealed the importance of the exchanges between local and foreign residents, including medical professionals, through Easy Japanese classes.

This study is subject to several limitations, including the fact that it is based on only three Easy Japanese sessions. The understanding of the current situation of the region and hospitals learned in this study session, as well as tips, issues, and points to keep in mind when using Easy Japanese in the medical field, are limited to generalization to other hospitals or communities. Further, participation by medical professionals was limited to a small group of students and professors at Shimane University School of Medicine. In addition, the interaction with "patients" is only a simulation, and practical research on "Easy Japanese" is also necessary. In the future, we would like to increase the number of participants and expand the study to include nursing students, medical students from other universities, and other medical professionals to confirm the usefulness of Easy Japanese for health communications, measure the improvement of individuals’ Easy Japanese skills, and promote the use of Easy Japanese among medical professionals.

## Conclusions

Currently, study sessions are being held mainly for medical students to make them aware of the existence of Easy Japanese communication. In addition, these sessions also help them understand the importance of expanding the means of communication and the usefulness for use in the medical field. On top of that, we measured the expansion of Easy Japanese communication skills and practiced it practically. So far, no Easy Japanese study group has been held at the hospital. In the future, the educational sessions of Easy Japanese need to increase the number of participants by including nursing students, medical students from various universities, and current medical professionals to confirm its usefulness in the medical field, improve individual skills, and promote the use of Easy Japanese among medical professionals.
